# Diagnosis and outcomes of acute kidney injury using surrogate and imputation methods for missing preadmission creatinine values

**DOI:** 10.1186/s12882-017-0552-3

**Published:** 2017-04-28

**Authors:** Amélie Bernier-Jean, William Beaubien-Souligny, Rémi Goupil, François Madore, François Paquette, Stéphan Troyanov, Josée Bouchard

**Affiliations:** 1 Department of Medicine, Division of Nephrology, Sacre-Coeur Hospital of Montreal, 5400 Gouin Blvd West, Montreal, Quebec H4J 1C5 Canada; 2 Faculty of Medicine, University of Montreal, Montreal, Quebec Canada

**Keywords:** Acute kidney injury, Baseline creatinine, Diagnosis, Epidemiology, Surrogate, Outcomes

## Abstract

**Background:**

Missing preadmission serum creatinine (SCr) values are a common obstacle to assess acute kidney injury (AKI) diagnosis and outcomes. The Kidney Disease Improving Global Outcomes (KDIGO) guidelines suggest using a SCr computed from the Modification of Diet in Renal Disease (MDRD) with an estimated glomerular filtration rate of 75 ml/min/1.73 m^2^. We aimed to identify the best surrogate method for baseline SCr to assess AKI diagnosis and outcomes.

**Methods:**

We compared the use of 1) first SCr at hospital admission 2) minimal SCr over 2 weeks after intensive care unit admission 3) MDRD computed SCr and 4) Chronic Kidney Disease Epidemiology Collaboration (CKD-EPI) computed SCr to assess AKI diagnosis and outcomes. We then performed multilinear regression models to predict preadmission SCr and imputation strategies to assess AKI diagnosis.

**Results:**

Our one-year retrospective cohort study included 1001 critically ill adults; 498 of them had preadmission SCr values. In these patients, AKI incidence was 25.1% using preadmission SCr. First SCr had the best agreement for AKI diagnosis (22.5%; kappa = 0.90) and staging (kappa = 0.81). MDRD, CKD-EPI and minimal SCr overestimated AKI diagnosis (26.7%, 27.1% and 43.2%;kappa = 0.86, 0.86 and 0.60, respectively). However, MDRD and CKD-EPI computed SCr had a better sensitivity than first SCr for AKI (93% and 94% vs. 87%). Eighty-eight percent of patients experienced renal recovery at least 3 months after hospital discharge. All methods except the first SCr significantly underestimated the percentage of renal recovery. In a multivariate model, age, male gender, hypertension, heart failure, undergoing surgery and log first SCr best predicted preadmission SCr (adjusted R^2^ = 0.56). Imputation methods with first SCr increased AKI incidence to 23.9% (kappa = 0.92) but not with MDRD computed SCr (26.7%;kappa = 0.89).

**Conclusion:**

In our cohort, first SCr performed better for AKI diagnosis and staging, as well as for renal recovery after hospital discharge than MDRD, CKD-EPI or minimal SCr. However, MDRD SCr and CKD-EPI SCr improved AKI diagnosis sensitivity. Imputation methods minimally increased agreement for AKI diagnosis.

**Electronic supplementary material:**

The online version of this article (doi:10.1186/s12882-017-0552-3) contains supplementary material, which is available to authorized users.

## Background

Acute kidney injury (AKI) is associated with higher mortality, longer hospital stays, and a higher likelihood of developing chronic kidney disease (CKD) [1–3]. AKI diagnosis relies on the quantification of changes in serum creatinine (SCr) from a preadmission value [4]*.* However, preadmission SCr is missing in 25–50% of patients [5–7], thereby being a major obstacle to accurately assess AKI diagnosis and outcomes, as recently highlighted by Siew and colleagues [8].

When baseline serum creatinine is missing, the Acute Dialysis Quality Initiative and Kidney Disease Improving Global Outcomes (KDIGO) guidelines suggest using a baseline SCr computed from the Modification of Diet in Renal Disease (MDRD) formula, assuming an estimated glomerular filtration rate (eGFR) of 75 ml/min per 1.73 m^2^ [4, 9]. This “ad hoc” suggestion was not a formal recommendation, due to the limited evidence on this issue*.* Some studies have assessed the performance of the MDRD or other surrogate methods for missing preadmission SCr for AKI diagnosis [5, 7, 10–17]. However, several factors other than age, race and gender may influence SCr levels, like comorbidities, fluid balance and prolonged hospital stay [18–20]. To our knowledge, only one study has attempted to improve the predictive performance of surrogate methods by including clinical characteristics into predictive models [12]. In that study, the full imputation method required variables not readily available, which may hinder its widespread use in clinical research.

In this study, our objectives were to 1) identify the most accurate surrogate method among those commonly used to estimate baseline SCr for AKI diagnosis and outcomes 2) identify variables associated with AKI diagnosis misclassification and 3) determine the value of imputation strategies to improve the capacity of estimating AKI diagnosis beyond surrogate methods.

## Methods

### Study design and participants

We performed a retrospective study of critically ill adult patients admitted to our tertiary care academic center between January 1^st^ and December 31^st^, 2012. In this study, we assessed the performance of four surrogate methods: 1) first SCr level at hospital admission; 2) minimal SCr level within 2 weeks after intensive care unit (ICU) admission; 3) SCr computed from the MDRD formula [21] for an eGFR of 75 ml/min per 1.73 m^2^ [4, 9] and 4) SCr computed from the Chronic Kidney Disease Epidemiology Collaboration (CKD-EPI) formula [22] for an eGFR of 75 ml/min per 1.73 m^2^. We performed a multilinear regression model to identify patients characteristics that best predict preadmission SCr. We then performed imputation strategies using calculated SCr values from the multilinear regression models to assess AKI diagnosis.

We included randomly selected critically ill patients aged 18 or more, and excluded readmissions, patients on chronic dialysis, those having a kidney transplant, or who stayed in the ICU less than 24 h. We followed the STrengthening the Reporting of OBservational studies in Epidemiology (STROBE) guidelines for observational studies [23].

### Data collection

We collected data on demographic characteristics, past medical history, laboratory results, severity of illness and processes of care from our ICU electronic medical record and by chart review. We retrieved SCr levels for each patient for up to one year before hospital admission. Outcomes included AKI diagnosis and staging, mortality at hospital discharge, and renal recovery at least 3 months after hospital discharge.

### Definitions

We used the KDIGO SCr criteria for AKI diagnosis and staging [4]; urine output criteria were not considered. AKI could be diagnosed if 1) there was an increase in serum creatinine (SCr) by ≥26.5 umol/l (≥0.3 mg/dl) within 48 h or 2) an increase in SCr to ≥1.5 times baseline, which is known or presumed to have occurred within the prior 7 days. We included creatinine values up to 48 h before ICU admission to allow for AKI assessment according to the first criterion. By definition, the 48-h criterion does not require a baseline creatinine value. We defined renal recovery by a decrease in SCr within 150% of baseline SCr. Preadmission or baseline SCr was defined as the closest value between 3 and 12 months before hospital admission to reflect CKD status [24], and if unavailable, the furthest value between 3 months before hospital admission and hospital admission. Preadmission SCr was considered missing if no SCr values were available before admission. Baseline eGFR was computed with MDRD using preadmission SCr, and CKD status was defined as an eGFR less than 60 ml/min per 1.73 m^2^ [24]. We also defined CKD status by examining medical records. Sequential Organ Failure Assessment (SOFA) scores were assessed at ICU admission [25]. Cumulative fluid balance was the sum of daily fluid balances during the first week of ICU admission.

### Statistical analyses and sample size calculation

Continuous variables are presented as mean ± standard deviation or median and interquartile range (IQR) and compared using *t*-test or Mann-Whitney U test, where appropriate. Categorical variables are presented as proportions and compared using *x* ^2^ test. We compared preadmission SCr and each surrogate method with the Bland-Altman method. For AKI diagnosis, we reported the different sensitivity and specificity for each surrogate method compared to preadmission SCr, and used the kappa statistic with 95% confidence intervals to report the level of agreement between the different surrogate methods and preadmission SCr. Misclassification rates were calculated as the proportion of patients incorrectly classified as AKI or non-AKI based on preadmission SCr, and we compared well-classified and misclassified AKI using the McNemar test. We reported similar statistics for AKI staging. We performed a multilinear regression analysis to predict preadmission SCr using variables associated with AKI misclassification and surrogate methods that had good predictive performance for AKI diagnosis. We used log-transformed creatinine for all SCr values. We then imputed the predicted SCr values from the different multilinear regression models (imputation strategy) to assess their effect on the incidence of AKI.

Statistical tests were two-sided and *p* values were reported. Appropriate adjustments for the Bonferroni correction were mentioned in tables with multiple *p* value comparisons. Statistical analyses were performed with SPSS, version 20.0 (IBM, Armonk, NY) and SAS 9.3 (SAS Institute, Cary, NC).

A sample size of 1073 subjects would achieve a 80% study power at a type 1 error of 0.05 to detect an absolute 5% difference in the incidence of AKI*.*

## Results

**Fig. 1:**
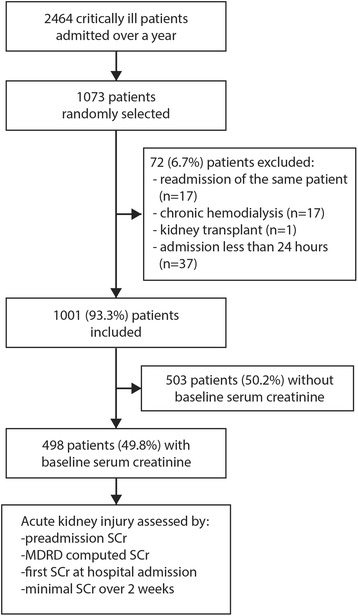
Study population

Among 2464 patients admitted over a year, 1073 (43.%) were randomly selected for the study population. From these, we excluded 17 patients (1.6%) who were readmitted over the same period, 17 patients on chronic hemodialysis (1.6%), one kidney transplant recipient (0.1%), and 37 patients who stayed in the ICU less than 24 h (3.4%) (Fig. 1). Among the remaining 1001 patients, 498 (50%) had a preadmission SCr value available and 121 (12%) suffered from CKD. Preadmission SCr were measured between 3 to 12 months prior to admission in 341 patients (68%), between 7 days to 3 months in 138 (28%), and less than 7 days prior to admission for 19 patients (4%).

### Characteristics of patients with and without preadmission creatinine

Table 1 included the demographic, comorbidities, and processes of care of the study population with and without available preadmission SCr. Out of 1001 patients, almost all were from Caucasian or Asian origin (97.1%, *n* = 972), 60.5% (*n* = 606) were male, and median age was 66 (IQR 56–75) years old. Patients with missing preadmission SCr were younger, less likely to suffer from chronic obstructive pulmonary disease (COPD), heart failure, cirrhosis, diabetes, CKD, cancer, underwent less surgical procedures but more often required mechanical ventilation. The severity of illness score, use of vasopressors and acute renal replacement therapy (RRT) were similar between groups, as well as lengths of stay (LOS) and mortality rates.

**Table 1:** Baseline characteristics of patients

	With preadmission creatinine (*n* = 498)	Without preadmission creatinine (*n* = 503)	*p*
Age (years) Gender (male %) Race (%)	67 (58–76) 59.4	65 (53–75) 61.6	0.005 0.48
Caucasian/Asian Body mass index Known preadmission creatinine (μmol/l)	96.4 27 (24–32) (*n* = 251) 73 (60–90)	97.8 27 (24–31) (*n* = 261) n/a	0.18 0.63
Baseline GFR MDRD (ml/min/1.73 m^2^)	89 (71–111)	n/a	
CAD (%) COPD (%) Heart failure (%) Cirrhosis (%) Diabetes (%) Hypertension (%) CKD by history (%) CKD MDRD <60 ml/min/1.73 m^2^ Cancer Surgery (%) SOFA score non-renal SOFA score Mechanical ventilation (%)	36.7 22.5 12.9 3.2 29.1 60.8 14.9 15.7 29.5 51.6 4 (2–7) 5 (2–8) 37.1	39.4 12.3 7.0 1.0 22.9 55.7 9.3 n/a 12.9 35.2 5 (2–8) 5 (2–8) 48.3	0.39 <0.001 0.002 0.02 0.02 0.10 0.007 <0.001 <0.001 0.08 0.21 <0.001
Vasopressors (%)	27.9	33.0	0.08
Cumulative fluid balance (L)	0.9 (-0.6 - 2.5)	0.8 (-0.7 - 2.4)	0.42
RRT	2.8	3.8	0.39
Hospital LOS Mortality	10 (6–21) 12.9	10 (6–23) 11.7	0.77 0.59

*CAD* coronary artery disease, *COPD* chronic obstructive pulmonary disease, *CKD* chronic kidney disease, *SOFA* Sequential Organ Failure Assessment Score, *RRT* renal replacement therapy, *LOS* length of stay

Data were missing in <1% of records

### Acute kidney injury diagnosis (*n* = 498)

The AKI incidence according to preadmission SCr was 25.1% (95% CI 21.5-29.1%). The first SCr method had the best agreement for AKI diagnosis (incidence of 22.5%, kappa = 0.90) (Table 2) but showed significant AKI misclassification with preadmission SCr (*p* < 0.004). Minimal SCr, MDRD and CKD-EPI computed SCr all overestimated AKI incidence (43.2%, 26.7%, 27.1%; kappa scores = 0.60, 0.86, and 0.86, respectively). Minimal SCr showed a significant misclassification rate (*p* < 0.0001) but not MDRD or CKD-EPI computed SCr methods. The MDRD and CKD-EPI methods were associated with a better sensitivity for AKI diagnosis than the first SCr (92.8% and 93.6% vs. 87.2%), at the expense of a slightly lower specificity (95.4% and 95.1% vs. 99.2%). We also compared preadmission SCr and each surrogate method with the Bland-Altman method (Additional file 1: Figures S1a-S1d).

**Table 2:** Acute kidney injury agreement between surrogate methods for missing baseline creatinine and preadmission serum creatinine

	AKI (%) *n* = 498	Sensitivity	Specificity	Kappa	McNemar*
Preadmission creatinine	25.1 (95% CI 21.5-29.1%; *n* = 125)	-	-	-	
First serum creatinine at hospital admission	22.5 (95% CI 19.0-26.4%; *n* = 112)	87.2	99.2	0.895 (95% CI 0.848-0.942)	0.004
Minimal creatinine within 2 weeks after ICU admission	43.2 (95% CI 38.9-47.6%; *n* = 215)	98.4	75.3	0.595 (95% CI 0.526-0.664)	<0.0001
MDRD computed serum creatinine	26.7 (95% CI 23.0-30.8%; *n* = 133)	92.8	95.4	0.864 (95% CI 0.813-0.915)	0.169
CKD-EPI computed serum creatinine	27.1 (95% CI 23.4-31.2; *n* = 135)	93.6	95.1	0.865 (95% CI 0.814-0.916)	0.076

*p < 0.01 represented significant difference, with the Bonferroni correction

Misclassification rates were 3.8% for the first SCr (overestimation 0.6% and underestimation 3.2%), 18.9% for the minimal SCr (overestimation 18.5% and underestimation 0.4%), 5.2% with MDRD (overestimation 3.4% and underestimation 1.8%), 5.2% with CKD-EPI (overestimation 3.6% and underestimation 1.6%)

MDRD: Modification of Diet in Renal Disease

CKD-EPI: Chronic Kidney Disease Epidemiology Collaboration

### Acute kidney injury staging (*n* = 498)

The first SCr was the most accurate method for AKI staging, with only 37 misclassified patients (7.4%) (Table 3). In comparison, using minimal SCr, 130 patients (26.1%) were misclassified (Table 4), with MDRD computed SCr, 61 patients (12.2%) (Table 5), and with CKD-EPI, 65 patients (13.1%) (Table 6). Thirteen patients required acute dialysis for AKI and were therefore classified as AKI stage 3, irrespective of SCr values, as defined by the KDIGO criteria [4]. When considering only moderate to severe AKI (stages 2 and 3) as AKI and stage 1 as non-AKI, the misclassification rates were 5.4% (*n* = 27) for the first SCr, 9.8% (*n* = 49) with minimal SCr, 7.8% (*n* = 39) with MDRD SCr, and 8.4% (*n* = 42) with CKD-EPI.

**Table 3:** Acute kidney injury staging according to first creatinine at hospital admission compared to preadmission creatinine

		Preadmission serum creatinine				
		No AKI	1	2	3	total
First SCr	No AKI	370	7	4	5	386
	1	3	64	10	1	78
	2	0	3	6	2	11
	3	0	0	2	21	23
	Total	373	74	22	29	498

Kappa 0.811 (95% CI 0.756-0.866)

Misclassification rate was 7.4% (overestimation 1.6% and underestimation 5.8%) - McNemar *p* = 0.02

*p* < 0.01 represented significant difference, with the Bonferroni correction

**Table 4:** Acute kidney injury staging according to minimal creatinine compared to preadmission creatinine

		Preadmission serum creatinine				
		No AKI	1	2	3	Total
Minimal Scr	No AKI	281	1	0	1	283
	1	80	52	7	3	142
	2	12	18	12	2	44
	3	0	3	3	23	29
	Total	373	74	22	29	498

Kappa 0.502 (95% CI 0.437-0.567)

Misclassification rate was 26.1% (overestimation 23.3% and underestimation 2.8%) - McNemar *p* < 0.0001

*p* < 0.01 represented significant difference, with the Bonferroni correction

**Table 5:** Acute kidney injury staging according to MDRD computed creatinine compared to preadmission creatinine

		Preadmission serum creatinine				
		No AKI	1	2	3	Total
MDRD Scr	No AKI	356	7	2	0	365
	1	15	47	7	1	70
	2	2	10	10	4	26
	3	0	10	3	24	37
	Total	373	74	22	29	498

MDRD: Modification of Diet in Renal Disease

Kappa 0.711 (95% CI 0.650-0.771)

Misclassification rate was 12.2% (overestimation 8.0% and underestimation 4.2%) – McNemar *p* = 0.05

*p* < 0.01 represented significant difference, with the Bonferroni correction

**Table 6:** Acute kidney injury staging according to CKD-EPI computed creatinine compared to preadmission creatinine

		Preadmission serum creatinine				
		No AKI	1	2	3	Total
CKD-EPI Scr	No AKI	355	7	1	0	363
	1	16	46	8	1	71
	2	2	11	8	4	25
	3	0	10	5	24	39
	Total	373	74	22	29	498

CKD-EPI: Chronic Kidney Disease Epidemiology Collaboration

Kappa 0.694 (95% CI 0.633-0.755)

Misclassification rate was 13.1% (overestimation 8.8% and underestimation 4.2%) – McNemar *p* = 0.04

*p* < 0.01 represented significant difference, with the Bonferroni correction

### Renal recovery and mortality rates

The AKI in-hospital mortality rate using preadmission SCr was 25.6% (*n* = 32/125; 95% CI 18.8-33.9%). There were no differences in AKI mortality rates between the different surrogate methods and preadmission SCr. The mortality rate with first SCr was 26.8% (*n* = 30/112; 95% CI 19.5-35.7%; *p* = 0.86), with minimal SCr, 20.9% (*n* = 45/215; 95% CI 16.0-26.9%; *p* = 0.32), with MDRD computed SCr, 27.1% (*n* = 36/133; 95% CI 20.2-35.2%; *p* = 0.79) and with CKD-EPI, 26.7% (*n* = 36/135; 95% CI 19.9-34.7%; p = 0.84).

After hospital discharge, according to preadmission SCr, 88.0% of AKI patients (95% CI 78.7-93.6%;*n* = 66/75, excluding *n* = 18 with missing data) recovered their kidney function after at least 3 months. The first SCr was the only method associated with a similar percentage of renal recovery rate compared to preadmission SCr (89.4% 95% CI 79.7-94.8%;*n* = 59/66 excluding *n* = 16 missing data;*p* = 0.79). All other methods were associated with a significantly lower percentage of renal recovery, namely minimal SCr (59.0%;95% CI 50.7-66.8%;*n* = 82/139 excluding *n* = 31 missing data;p < 0.001), MDRD (63.3%;95% CI 52.3-73.1%;*n* = 50/79 excluding *n* = 18 missing data;*p* < 0.001) and CKD-EPI (59.3%;95% CI 48.4-69.3%;*n* = 48/81 excluding *n* = 18 missing data;*p* < 0.001).

### Clinical characteristics associated with misclassified AKI

We compared well-classified and misclassified AKI by the surrogate method with the highest level of agreement, i.e. the first SCr at hospital admission. Misclassified patients were more likely to be female, and to suffer from CKD. They had a higher SOFA score, used vasopressors more frequently, were less likely to suffer from hypertension or undergo surgery (Additional file 2: Table S1a). We also compared well-classified and misclassified AKI by MDRD computed SCr (Additional file 2: Table S1b). Misclassified patients were more likely to suffer from heart failure, diabetes, and CKD, and had a higher SOFA score, while they were less likely to suffer from COPD, cancer or undergo surgery. We further detailed misclassified AKI by missed and overdiagnosed AKI in Additional file 2: Table S1c and d.

We also assessed whether cumulative fluid balance and ICU length of stay were different between well-classified and misclassified AKI using the minimal SCr, as positive fluid balance and longer ICU stay may affect subsequent SCr levels and therefore, AKI diagnosis [18–20]. Neither cumulative fluid balance nor ICU length of stay was significantly associated with AKI misclassification using minimal SCr.

### Multilinear regression model and imputation strategies

We included in our multilinear regression model clinical characteristics that were associated with misclassified AKI rates (Additional file 2: Table S1a and S1b) and surrogate methods that had a good predictive capacity for AKI diagnosis (first SCr and MDRD computed SCr). We did not include vasopressors as they are part of SOFA scores, and adjusted the results for age and gender. Age, male gender, hypertension, heart failure, undergoing surgery and log first SCr best predicted preadmission SCr (adjusted R^2^ = 0.56) (Table 7). Using log MDRD computed SCr, age, hypertension, and heart failure were still included in the final model, as well as diabetes and SOFA score (Table 7). However, the model did not perform as well as with log first SCr (adjusted R^2^ = 0.28).

**Table 7:** Multiple regression analysis to predict preadmission serum creatinine

	Dependent Variable Log (preadmission SCr)	
Covariates or factor included in model	Model 1	Model 2
Constant	0.844 (0.049)	-3.728 (2.400)
Log (first SCr) Log (MDRD computed SCr) Age Male gender	0.461^a^ (0.026) n/a 0.001^b^ (0.000) 0.061^a^ (0.010)	n/a 2.744^e^ (1.233) 0.006^a^ (0.001) -0.209 (0.139)
Hypertension COPD	0.029^c^ (0.011) -0.011 (0.012)	0.048^a^ (0.014) -0.005 (0.015)
Heart failure	0.037^d^ (0.015)	0.099^a^ (0.019)
Diabetes	0.014 (0.011)	0.034^d^ (0.014)
Cancer Surgery SOFA score Observations *R* ^*2*^ *Adjusted R* ^*2*^	0.015 (0.011) 0.027^a^ (0.010) -0.001 (0.001) 498 0.57 0.56	0.020 (0.014) -0.018 (0.013) 0.006^b^ (0.002) 498 0.30 0.28

Regression coefficients for covariates or factor (SEM in parentheses)

^a^ *p* ≤ 0.001. ^b^ *p* = 0.002. ^c^ *p* = 0.01. ^d^ *p* = 0.02. ^e^ *p* = 0.03

*SCr* serum creatinine, *MDRD* Modification of Diet in Renal Disease, *COPD* chronic obstructive pulmonary disease, *SOFA* Sequential Organ Failure Assessment score

We then assessed whether there would be improvement in AKI classification with predicted SCr from multilinear regression models. When using SCr obtained from the first SCr model, the AKI incidence increased to 23.9% (kappa = 0.92; 95% CI 0.89-0.96). When using SCr obtained from the MDRD SCr model, the incidence of AKI remained unchanged (26.7%; kappa = 0.89; 95% CI 0.84-0.93).

## Discussion

Missing preadmission SCr values are a common obstacle in AKI research. The use of surrogate methods for baseline SCr allows researchers to avoid selection bias, but could lead to inaccurate AKI diagnosis and prognosis. In 2004, the Acute Dialysis Quality Initiative (ADQI) group suggested using a surrogate computed SCr from an eGFR of 75 ml/min per 1.73 m^2^ for missing baseline SCr based on expert opinions [9]. However, it is not entirely clear what is recommended if the first SCr at hospital admission is lower than the eGFR of 75 ml/min per 1.73 m^2^ estimate. Despite an increasing number of AKI studies published over the last decade [26–31], only a few have evaluated the clinical relevance of using different surrogate baseline SCr to assess AKI diagnosis and outcomes [5, 7, 10–17].

We report our findings using four different surrogate methods (MDRD, CKD-EPI, minimal and first SCr) and imputation strategies to estimate preadmission SCr for AKI diagnosis. In our study, preadmission SCr was missing in 50% of patients, which is similar to previous results from the literature [5–7, 15]. Patients without preadmission SCr are less likely to have medical follow-up and may be healthier than those with medical follow-up. As expected, we found that patients without preadmission SCr were younger and suffered from less comorbidities including CKD (by chart review) than patients with available preadmission SCr.

To our knowledge, our study is the first to simultaneously compare these four methods for estimating baseline SCr for AKI diagnosis. Our results show that measures to estimate baseline SCr either underestimate or overestimate AKI incidence, which could affect outcomes associated with presumed AKI. In our population, the AKI incidence was 25.1% with preadmission SCr compared to 22.5% using the first SCr, 26.7% with MDRD, 27.1% with CKD-EPI, and 43.2% with the minimal SCr. The first SCr at hospital admission, CKD-EPI and MDRD methods had very good agreement scores with preadmission SCr to diagnose AKI. The minimal SCr within two weeks after ICU admission had only moderate agreement. Previous studies have also shown that the use of first SCr can decrease AKI incidence [11, 15] and lower sensitivity for AKI diagnosis. These findings have clinical implications since failure to diagnose AKI with lack of preventive and therapeutic measures is different than overdiagnosing AKI and provide unnecessary treatments. Similar to our findings, a few studies have shown that the MDRD method tends to overestimate the incidence of AKI [5, 7, 10, 11, 14, 15]. Two studies have shown that the CKD-EPI overestimates AKI incidence [10, 17]. The magnitude of the overestimation is related to the prevalence of CKD. In our study, the baseline eGFR (89 ml/min per 1.73 m^2^) was either similar [10, 14, 26] or higher [5, 11, 12] than in other studies, which improved the ability of the MDRD and CKD-EPI methods to accurately diagnose AKI. Importantly, we found that the minimal SCr within two weeks after ICU admission markedly overestimated the incidence of AKI and thereby reduced its specificity. SCr values from the minimal SCr method were more often lower than the preadmission SCr, possibly due to fluid administration, decreased creatinine production or cessation of renin– angiotensin–aldosterone system blockers [18, 19, 32]. Some studies have assessed the performance of different definitions of minimal SCr on AKI diagnosis with conflicting results [10, 11, 16]. In practice, the use of minimal inpatient SCr for AKI diagnosis is not ideal, as the diagnosis of AKI can only be made retrospectively. Importantly, to our knowledge, the only study that has simultaneously compared first SCr, MDRD and minimal SCr for AKI diagnosis showed comparable trends regarding the overestimation or underestimation of AKI for each method [11].

Regarding AKI staging, the first SCr at hospital admission provided better accuracy than MDRD computed SCr in our population, with a misclassified AKI staging rate of 7.4%. The MDRD misclassified 12.2% of AKI stages, CKD-EPI, 13.1%, and the minimal SCr, 26.1%. Siew and colleagues also showed that the first SCr performs better for AKI staging than MDRD and minimal SCr [11]. Pickering and colleagues found similar misclassified rate with MDRD SCr than our study (11.1%) [10]. The rates of misclassification found by Siew and colleagues were higher than in our study [11]. Differences in the prevalence of CKD and timing or severity of AKI severity partly explain these results. A higher proportion of CKD will increase misclassification associated with MDRD SCr and CKD-EPI SCr, while AKI at hospital admission will increase misclassification with the first SCr method. A higher need for RRT will reduce misclassification with all different surrogate methods, as these will all be categorized as AKI stage 3.

To our knowledge, our study is the first to assess the performance of four different surrogate methods on the rates of renal recovery after hospital discharge. Assessing renal recovery after hospital discharge avoids systematic bias related to decreases in SCr after critical illness that persist until hospital discharge [33]. Using preadmission SCr, we found that 88.0% of AKI patients had renal recovery after hospital discharge. The first SCr was the only surrogate method not associated with a significantly lower performance for renal recovery (89.4%) compared to preadmission SCr. The minimal, MDRD and CKD-EPI computed SCr methods all significantly underestimated renal recovery. As the MDRD and CKD-EPI computed SCr will underestimate the preadmission SCr in CKD, the rates of renal recovery are expected to be lower with these surrogate methods. More importantly, the percentage of non-renal recovery will be higher as the percentage of CKD patients increases. The MDRD and CKD-EPI surrogate methods should not be used to estimate renal recovery in populations with high prevalence of CKD. The minimal SCr was associated with a lower renal recovery rate possibly due to decreased muscle mass and falsely low SCr values during hospital stay [33]. Our study was underpowered to assess differences in mortality and showed similar percentages of mortality rates for the different surrogate methods. Nevertheless, there were some misclassifications of AKI deaths which could reduce the validity of risk-prediction models for AKI mortality.

It is well known that GFR decreases with age and comorbidities such as long-term diabetes and hypertension and older age [34–36]. In our predictive models with first SCr or MDRD computed SCr, older age and comorbidities like hypertension and heart failure improved the prediction of baseline SCr. Being male or suffering from diabetes also helped to predict baseline SCr with first SCr or MDRD computed SCr, respectively. These results were expected as these variables are known to be associated with a higher likelihood of CKD. We also found that a higher SOFA scores helped to predict baseline SCr from MDRD computed SCr, and being admitted for surgery helped to predict baseline SCr from the first SCr. These results may reflect underlying comorbidities associated with these findings. Imputation methods minimally increased the agreement for AKI diagnosis.

Our study has several strengths. We included critically ill patients from specialized intensive care units (cardiac surgery, neurosurgery/neurological, trauma, medical) with detailed data on patient characteristics and had limited exclusion criteria to increase generalizability. We assessed the performance of four commonly used surrogate methods not only for AKI diagnosis and staging, but also for renal recovery and mortality. Our study also has limitations. The pattern of missing preadmission SCr and percentage of CKD in our academic center may not be similar in other centers, and baseline kidney function may be different between patients with and without baseline creatinine, affecting AKI diagnosis by estimating methods. However, we found a similar percentage of missing baseline SCr [12] and comparable baseline eGFR [10, 14, 29] in other large epidemiological AKI studies. Also, almost all patients were Caucasian or Asian. Therefore, our results may not be applicable to patients from other races. This study was performed in critically ill patients and results may be different in elective surgical patients or those admitted to hospital wards. We chose to define baseline SCr as the closest value between 3 and 12 months before admission to reflect CKD diagnosis by the KDIGO [24], and if unavailable, the value furthest to 90 days before admission. Other studies have chosen SCr values 7–365 days before admission which may affect overall results [15, 37]. Finally, with a larger database, we might have shown statistically different mortality rates between surrogate methods and preadmission SCr in AKI patients.

## Conclusion

Our results have important research and clinical implications. The use of different surrogate methods can significantly affect AKI incidence and outcomes. In our cohort, the first SCr had a better agreement for diagnosing and staging AKI than MDRD, CKD-EPI and minimal SCr. Our results concur with the recommendation from the European Renal Best Practice position statement on the KDIGO guidelines to use the first SCr of the episode when baseline SCr is missing [38]. However, MDRD SCr and CKD-EPI improved AKI diagnosis sensitivity compared to first SCr in our population having a relatively low prevalence of CKD (16%). Importantly, even with a low prevalence of CKD, MDRD and CKD-EPI SCr significantly underestimated renal recovery after hospital discharge compared to other methods. Therefore, research using MDRD or CKD-EPI computed SCr as surrogate methods for missing baseline SCr should be interpreted with caution in populations with a higher prevalence of CKD. Similarly, the use of minimal SCr during ICU stay also underestimates renal recovery after hospital discharge. Our multilinear regression model identified several variables helping to predict baseline SCr. However, imputation methods only minimally increased agreement for AKI diagnosis. Further research is needed on the effect of using surrogate methods and imputation methods for baseline SCr on AKI diagnosis and outcomes.

## Additional files

Additional file 1: Figure S1a-1d. Bland-Altman methods between preadmission serum creatinine and various surrogates methods for estimating baseline serum creatinine. (a) first serum creatinine, (b) minimal serum creatinine, (c) MDRD computed serum creatinine, (d) CKD-EPI computed serum creatinine. SCr: serum creatinine; MDRD: Modification of Diet in Renal Disease; CKD-EPI: Chronic Kidney Disease Epidemiology Collaboration. (PNG 78 kb)

Additional file 2: **Table S1a.** Comparison between well-classified and misclassified acute kidney injury by the first serum creatinine method. **Table S1b.** Comparison between well-classified and misclassified acute kidney injury by the Modification of Diet in Renal Disease (MDRD) method. **Table S1c.** Comparison between well-classified, missed and overdiagnosed acute kidney injury by the first serum creatinine method. **Table S1d.** Comparison between well-classified, missed and overdiagnosed acute kidney injury by the Modification of Diet in Renal Disease (MDRD) method. (DOCX 87 kb)

## Abbreviations

AKI: acute kidney injury
AQQI: Acute dialysis quality initiative
CAD: coronary artery disease
CKD: chronic kidney disease
CKD-EPI: Chronic kidney disease epidemiology collaboration
COPD: chronic obstructive pulmonary disease
eGFR: estimated glomerular filtration rate
ICU: intensive care unit
IQR: interquartile range
KDIGO: Kidney disease improving global outcomes
LOS: lengths of stay
MDRD: Modification of diet in renal disease
RRT: renal replacement therapy
SCr: serum creatinine
SOFA: Sequential organ failure assessment
STROBE: STrengthening the reporting of observational studies in epidemiology
