# Supplementary figures and images for: Diagnosis and outcomes of acute kidney injury using surrogate and imputation methods for missing preadmission creatinine values
Source: BMC Nephrol. 2017 Apr 28;18:141. doi: 10.1186/s12882-017-0552-3 (PMC5410063; doi:10.1186/s12882-017-0552-3)

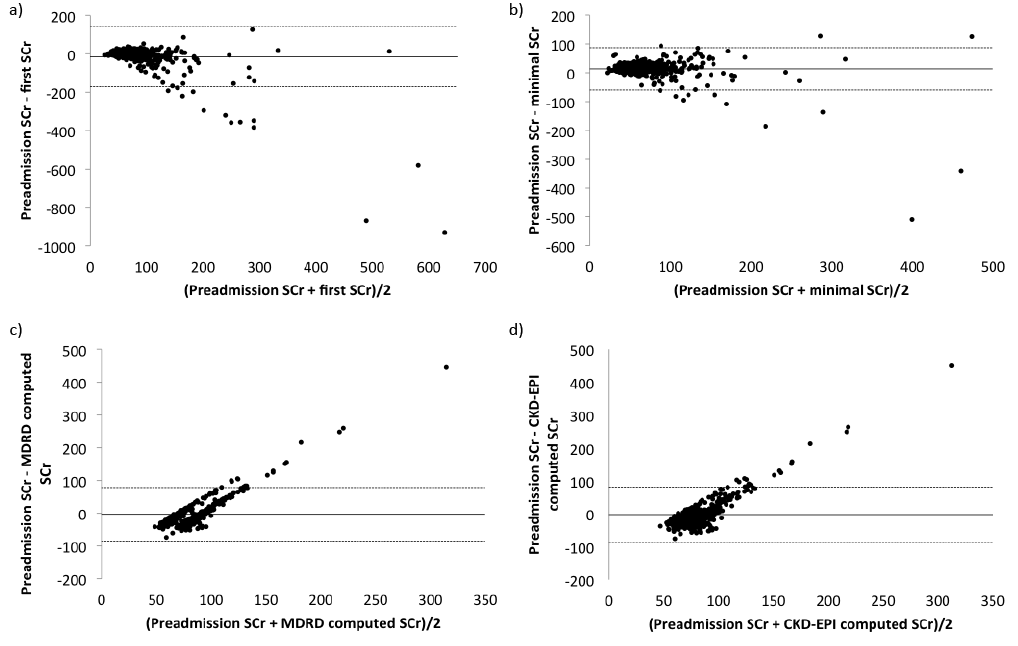

Supplement: Supplementary file 1 — Bland-Altman methods between preadmission serum creatinine and various surrogates methods for estimating baseline serum creatinine. (a) first serum creatinine, (b) minimal serum creatinine, (c) MDRD computed serum creatinine, (d) CKD-EPI computed serum creatinine. SCr: serum creatinine; MDRD: Modification of Diet in Renal Disease; CKD-EPI: Chronic Kidney Disease Epidemiology Collaboration. (PNG 78 kb) [file 12882_2017_552_MOESM1_ESM.png]
