# Supplementary material for: Diagnosis and outcomes of acute kidney injury using surrogate and imputation methods for missing preadmission creatinine values
Source: BMC Nephrol. 2017 Apr 28;18:141. doi: 10.1186/s12882-017-0552-3 (PMC5410063; doi:10.1186/s12882-017-0552-3)
Supplement: Supplementary file 2 — Table S1a. Comparison between well-classified and misclassified acute kidney injury by the first serum creatinine method. Table S1b. Comparison between well-classified and misclassified acute kidney injury by the Modification of Diet in Renal Disease (MDRD) method. Table S1c. Comparison between well-classified, missed and overdiagnosed acute kidney injury by the first serum creatinine method. Table S1d. Comparison between well-classified, missed and overdiagnosed acute kidney injury by the Modification of Diet in Renal Disease (MDRD) method. (DOCX 87 kb) [file 12882_2017_552_MOESM2_ESM.docx]

**Supplement Table 1a. Comparison between well-classified and misclassified acute kidney injury by the first serum creatinine method**

|  | AKI well classified by first SCr  (n=479) | AKI misclassified  by first SCr  (n=19) | P |
| --- | --- | --- | --- |
| Age (years)  Gender (male %)  Race (%)  Caucasian/Asian  Known preadmission creatinine (µmol/l)  Baseline GFR MDRD (ml/min/1.73 m^2^)  CAD (%)  COPD (%)  Heart failure (%)  Cirrhosis (%)  Diabetes (%)  Hypertension (%)  CKD by history (%)  CKD MDRD <60 ml/min/1.73 m^2^ (%)  Cancer (%)  Surgery (%)  SOFA score non-renal  SOFA score  Mechanical ventilation (%)  Vasopressors (%)  Cumulative fluid balance (L)  RRT (%)  Hospital LOS (days)  Mortality (%) | 67 (58-76)  60.3  96.7  73 (61-90)  88 (71-109)  37.4  22.5  12.3  3.1  29.4  61.8  14.2  15.7  30.1  53.0  4 (2-7)  5 (2-8)  38.0  26.9  0.9 (-0.6 - 2.5)  2.9  10 (6-21)  12.9 | 72 (53-77)  36.8  89.5  59 (50-84)  109 (80-131)  21.1  21.1  26.3  5.3  21.1  36.8  31.6  15.8  15.8  15.8  6 (4-7)  7 (5-9)  15.8  52.6  2.0 (-0.8 – 3.0)  0  16 (10-24)  10.5 | 0.44  0.04  0.15  0.02  0.06  0.15  0.88  0.07  0.47  0.43  0.03  0.04  0.99  0.21  0.001  0.02  0.005  0.05  0.01  0.30  1.0  0.12  0.76 |

**Legend:** CAD: coronary artery disease; COPD: chronic obstructive pulmonary disease; CKD: chronic kidney disease; SOFA: Sequential Organ Failure Assessment Score; RRT: renal replacement therapy; LOS: length of stay

Data were missing in <1% of records.

**Supplement Table 1b. Comparison between well-classified and misclassified acute kidney injury by the Modification of Diet in Renal Disease (MDRD) method**

|  | AKI well classified by MDRD  (n=472) | AKI misclassified  By MDRD  (n=26) | P |
| --- | --- | --- | --- |
| Age (years)  Gender (male %)  Race (%)  Caucasian/Asian  Known preadmission creatinine (µmol/l)  Baseline GFR MDRD (ml/min/1.73 m^2^)  CAD (%)  COPD (%)  Heart failure (%)  Cirrhosis (%)  Diabetes (%)  Hypertension (%)  CKD by history (%)  CKD MDRD <60 ml/min/1.73 m^2^ (%)  Cancer (%)  Surgery (%)  SOFA score non-renal  SOFA score  Mechanical ventilation (%)  Vasopressors (%)  Cumulative fluid balance (L)  RRT (%)  Hospital LOS (days)  Mortality (%) | 67 (58-76)  59.5  96.6  72 (60-88)  89 (72-111)  36.2  23.5  11.9  3.0  28.0  60.2  13.3  13.8  30.5  53.2  4 (2-7)  5 (2-8)  37.3  27.3  0.9 (-0.6 - 2.5)  3.0  10 (6-21)  12.3 | 75 (59-80)  57.7  92.3  107 (56-142)  60 (39-113)  46.2  3.8  30.8  7.7  50.0  73.1  42.3  50.0  11.5  23.1  6 (3-8)  7 (4-9)  34.6  38.5  0.5 (-1.9 - 2.9)  0  10 (6-26)  23.1 | 0.10  0.85  0.24  0.02  0.008  0.31  0.02  0.01  0.20  0.02  0.19  <0.001  <0.001  0.04  0.003  0.21  0.03  0.78  0.22  0.49  1.0  0.96  0.13 |

**Legend:** CAD: coronary artery disease; COPD: chronic obstructive pulmonary disease; CKD: chronic kidney disease; SOFA: Sequential Organ Failure Assessment Score; RRT: renal replacement therapy; LOS: length of stay

Data were missing in <1% of records.

**Supplement Table 1c. Comparison between well-classified, missed and overdiagnosed acute kidney injury by the first serum creatinine method**

|  | AKI well classified by first SCr  (n=479) | AKI missed  by first SCr  (n=3) | AKI overdiagnosed  by first SCr  (n=16) | P |
| --- | --- | --- | --- | --- |
| Age (years)  Gender (male %)  Race (%)  Caucasian/Asian  Known preadmission creatinine (µmol/l)  Baseline GFR MDRD (ml/min/1.73 m^2^)  CAD (%)  COPD (%)  Heart failure (%)  Cirrhosis (%)  Diabetes (%)  Hypertension (%)  CKD by history (%)  CKD MDRD <60 ml/min/1.73 m^2^ (%)  Cancer (%)  Surgery (%)  SOFA score non-renal  SOFA score  Mechanical ventilation (%)  Vasopressors (%)  Cumulative fluid balance (L)  RRT (%)  Hospital LOS (days)  Mortality (%) | 67 (58-76)  60.3  96.7  73 (61-90)  88 (71-109)  37.4  22.5  12.3  3.1  29.4  61.8  14.2  15.7  30.1  53.0  4 (2-7)  5 (2-8)  38.0  26.9  0.9 (-0.6 - 2.5)  2.9  10 (6-21)  12.9 | 73 (50-)  33.3  66.7  59 (50-)  111 (90-)  33.3  33.3  33.3  0.0  33.3  33.3  0.0  0.0  0.0  66.7  6 (6-)  6 (6-)  33.3  33.3  2.7 (1.4-)  0.0  10 (9-)  0.0 | 72 (56-77)  37.5  93.8  59 (50-86)  108 (74-132)  18.8  18.8  25.0  6.3  18.8  37.5  37.5  18.8  18.8  6.3  6 (4-8)  8 (5-10)  12.5  56.3  1.6 (-1.0 – 3.7)  0.0  17 (11-26)  12.5 | 0.57  0.12  0.02  0.05  0.14  0.31  0.85  0.19  0.75  0.64  0.09  0.03  0.71  0.33  0.001  0.19  0.03  0.12  0.04  0.58  0.75  0.14  0.80 |

**Legend:** CAD: coronary artery disease; COPD: chronic obstructive pulmonary disease; CKD: chronic kidney disease; SOFA: Sequential Organ Failure Assessment Score; RRT: renal replacement therapy; LOS: length of stay

Data were missing in <1% of records.

**Supplement Table 1d. Comparison between well-classified, missed and overdiagnosed acute kidney injury by the Modification of Diet in Renal Disease (MDRD) method**

|  | AKI well classified by MDRD  (n=472) | AKI missed  by MDRD  (n=17) | AKI overdiagnosed  by MDRD  (n=9) | P |
| --- | --- | --- | --- | --- |
| Age (years)  Gender (male %)  Race (%)  Caucasian/Asian  Known preadmission creatinine (µmol/l)  Baseline GFR MDRD (ml/min/1.73 m^2^)  CAD (%)  COPD (%)  Heart failure (%)  Cirrhosis (%)  Diabetes (%)  Hypertension (%)  CKD by history (%)  CKD MDRD <60 ml/min/1.73 m^2^ (%)  Cancer (%)  Surgery (%)  SOFA score non-renal  SOFA score  Mechanical ventilation (%)  Vasopressors (%)  Cumulative fluid balance (L)  RRT (%)  Hospital LOS (days)  Mortality (%) | 67 (58-76)  59.5  96.6  72 (60-88)  89 (72-111)  36.2  23.5  11.9  3.0  28.0  60.2  13.3  13.8  30.5  53.2  4 (2-7)  5 (2-8)  37.3  27.3  0.9 (-0.6 - 2.5)  3.0  10 (6-21)  12.3 | 76 (68-83)  70.6  94.1  129 (107-160)  41 (36-60)  55.8  59.9  47.1  5.9  64.7  88.2  64.7  3.4  11.8  35.3  6 (3-9)  7 (4-10)  41.2  47.1  0.6 (-2.3 – 3.0)  0.0  8 (5-29)  29.4 | 56 (40-78)  33.3  88.9  45 (39-62)  141 (103-170)  22.2  0.0  0.0  11.1  22.2  44.4  0.0  0.0  11.1  0.0  5 (4-6)  6 (4-7)  22.2  22.2  0.2 (-2.7 – 4.8)  0.0  13 (8-30)  11.1 | 0.16  0.18  0.41  <0.00  <0.001  0.11  0.06  <0.001  0.32  0.004  0.04  <0.001  <0.001  0.12  0.003  0.009  <0.001  0.61  0.19  0.88  0.67  0.34  0.12 |

**Legend:** CAD: coronary artery disease; COPD: chronic obstructive pulmonary disease; CKD: chronic kidney disease; SOFA: Sequential Organ Failure Assessment Score; RRT: renal replacement therapy; LOS: length of stay

Data were missing in <1% of records.
